# Understanding the complex relationship between amino acid absorption kinetics and postprandial muscle protein synthesis rates in healthy adults and critically ill patients

**DOI:** 10.1097/MCO.0000000000001181

**Published:** 2025-11-06

**Authors:** Oliver C. Witard, Konstantinos Prokopidis, Colleen S. Deane

**Affiliations:** a Centre for Human and Applied Physiological Sciences, Faculty of Life Sciences and Medicine, King's College London, London; b Department of Musculoskeletal and Ageing Science, Institute of Life Course and Medical Sciences, University of Liverpool, Liverpool; c Human Development & Health, Faculty of Medicine, University of Southampton, Southampton General Hospital; d NIHR Southampton Biomedical Research Centre, University Hospital Southampton, Southampton, UK

**Keywords:** protein digestibility, amino acid kinetics, leucine trigger, muscle protein turnover, critical illness

## Abstract

**Purpose of review:**

Protein digestion and amino acid absorption kinetics are quantifiable metrics commonly utilized to determine the quality of a protein source. This review critically evaluates recent evidence (primarily from studies that provided commonly consumed protein-rich foods) regarding the relationship between *in vivo* protein digestion and amino acid absorption rates with the postprandial stimulation of muscle protein synthesis (MPS), with an emphasis on healthy adults and critically ill patients.

**Recent findings:**

Ingested protein sources that elicit moderate amino acid bioavailability, including leucine, stimulate MPS rates to a comparable extent as protein sources that elicit high amino acid bioavailability in healthy young adults. Amino acid absorption kinetics appear to be modulated in critically ill patients, leading to a marked reduction in postprandial MPS rates. Preliminary studies demonstrate that enteral feeding of high dose free amino acids increase amino acid bioavailability to a greater extent than intact protein, leading to a positive whole-body net protein balance in critically ill patients. However, in practice, the high osmolarity of free amino acids leads to a high prevalence of diarrhoea and thus limits the clinical application of this intervention.

**Summary:**

The enteral provision of free amino acids represents a theoretical, but not practically-relevant, clinical nutrition strategy to mitigate the catabolic response to critical illness. Future studies are warranted to establish targeted protein/amino acid-based interventions to mitigate skeletal muscle atrophy during the metabolic care of critically ill patients.

## INTRODUCTION

Dietary protein-derived amino acids provide the primary substrate and signal for muscle protein synthesis (MPS) and thus are fundamental in regulating skeletal muscle mass across the healthspan. Accordingly, the concept of dietary protein quality continues to receive scientific attention with regards to informing protein guidelines across clinical nutrition and metabolic care settings [1]. Protein quality is fundamentally determined by the essential amino acid (EAA) composition and digestibility of a given protein source, and subsequent amino acid absorption from the gastrointestinal lumen in the small intestine [2]. The rate of protein digestion and amino acid absorption determines the postprandial rise in circulating amino acids and is widely recognized to modulate postprandial MPS rates, although this relationship has recently been considered more complex than is often espoused [3,4]. Contemporary evidence also indicates that amino acid absorption rates may be compromised in critical illness [5], with implications for personalized protein nutrition recommendations in critically ill patients [6]. This review synthesizes recent evidence regarding the complex relationship of protein digestion and amino acid absorption rates with the postprandial stimulation of MPS, with specific reference to studies conducted in healthy adults and critically ill patients. 

**Box 1:**
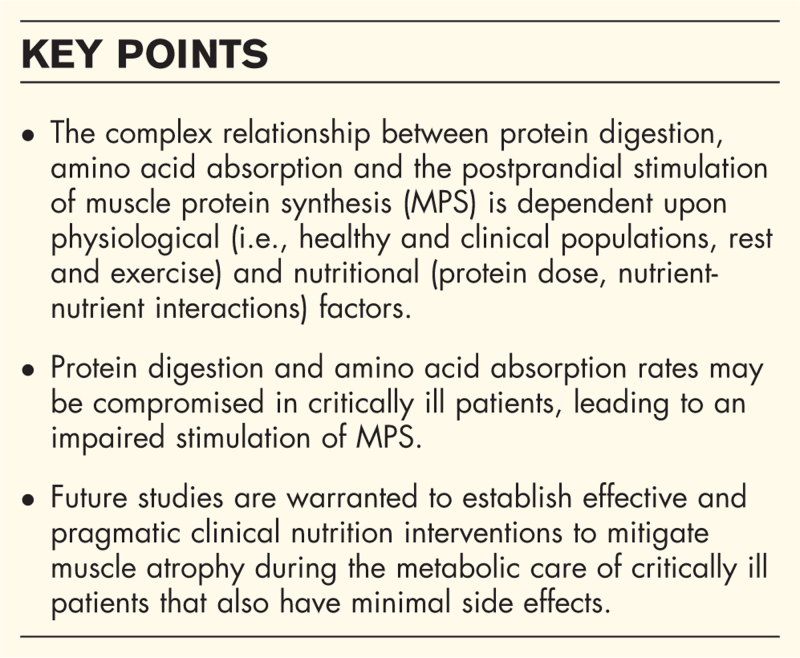
no caption available

## TERMINOLOGY

Whilst dietary protein digestion and amino acid absorption kinetics are firmly established as primary factors in determining the quality of a protein source [7], there remains confusion and a lack of precision around relevant terminology [8]. The term ‘protein digestion’ is defined as the mechanical and chemical breakdown of protein into amino acids within the gastrointestinal (GI) tract, whereas ‘amino acid absorption’ describes the process of amino acid uptake from the GI lumen [8]. Accordingly, the term ‘protein digestibility’ refers to the proportion of ingested dietary protein that is broken down into absorbable constituents for the GI tract, and ‘amino acid absorbability’ describes the proportion of amino acids that are taken up from the GI lumen [8]. In addition, ‘protein bioavailability’ encapsulates the proportion of ingested protein-derived amino acids (also dipeptides and tripeptides) absorbed into the circulation that renders amino acids available as substrate for the postprandial stimulation of MPS [8]. Finally, the ‘postprandial handling of dietary protein’ is increasingly recognized as an all-encompassing term used to describe the sequence of metabolic processes pertaining to the digestion, absorption and subsequent stimulation of MPS in response to an ingested protein source [9]. The consistent use of precise terminology is critical in advancing understanding into the role of protein quality in clinical nutrition.

To achieve a comprehensive assessment of postprandial protein handling *in vivo* in humans, intravenous stable isotope-labelled amino acid infusions can be combined with the ingestion of an intrinsically-labelled protein [9]. This tandem approach enables the simultaneous measurement of protein digestion and amino acid absorption kinetics, alongside informative physiological readouts such as the postprandial stimulation of MPS [10]. Largely attributed to the expensive monetary cost and labour-intensive nature of this oral-intravenous dual tracer method, our understanding of postprandial protein handling for a range of protein sources under a variety of conditions and within different clinical populations continues to emerge, albeit at a steady rate. Nonetheless, based on recent findings, the importance of protein digestion and amino acid absorption kinetics as independent determinants of protein quality may have been over-simplified within a clinical nutrition setting.

## PROTEIN DIGESTION AND AMINO ACID ABSORPTION KINETICS IN HEALTHY ADULTS

Current opinion regarding the nutritional profile of a high-quality protein source is rapidly evolving. Traditionally, the rate of protein digestion and amino acid absorption that determines the postprandial rise in circulating amino acids is widely recognized to modulate the postprandial stimulation of MPS [11]. Specifically, it was proposed that a directly proportional positive association exists between the amplitude and rate of postprandial increase in blood leucine concentration, and the magnitude of the MPS response to an ingested protein source. This phenomenon was coined as the ‘leucine trigger’ hypothesis [12] and is underpinned by robust molecular studies that highlight the potency of leucine (vs. other EAA's) in upregulating the mechanistic target of rapamycin complex 1 (mTORC1) signalling cascade that activates the translation initiation step of MPS [13]. In comparison, the ‘leucine threshold’ concept quantifies the minimum leucine availability required for the intracellular machinery to stimulate MPS [14^▪^]. Whilst the leucine trigger hypothesis provides valuable mechanistic insight regarding the postprandial regulation of *in vivo* MPS rates in humans, from an applied perspective, we [3] and others [4] have recently highlighted the complex nature of applying the leucine trigger hypothesis across all physiological scenarios and populations. This more cautious approach is primarily based on emerging evidence that, under certain conditions, a dissociation exists between ingested leucine or plasma leucine concentrations and the magnitude of postprandial MPS rates in response to ingested protein [14^▪^].

A consistent [15–20], albeit not universal [21], finding across a series of recent experimental studies conducted in healthy young and older adults concerns the apparent disconnect between blood leucine concentration profiles and postprandial MPS rates in response to a variety of ingested protein sources, that is, crystalline free amino acids, plant and animal proteins, and alternative (fungal, algal, insect) proteins (Table 1). For instance, the ingestion of crystalline free amino acids compared with an equivalent 30 g dose of intact milk protein resulted in a more rapid amino acid absorption and greater postprandial plasma amino acid availability, albeit without affecting corresponding MPS rates in healthy young adults [15]. This disassociation between plasma leucine concentrations and postprandial MPS rates has also consistently been reported across a recent programme of studies conducted at Maastricht University that compared various isolated intact plant proteins, namely wheat hydrolysate [18], pea concentrate [17], potato concentrate [22], corn isolate [23] or a blend of wheat hydrolysate, corn isolate and pea concentrate protein [24], with a dose-matched milk concentrate control condition in healthy young adults. Indeed, while the increase in plasma leucine concentration was less pronounced after ingesting 30 g of pea protein vs. 30 g of milk protein, postprandial MPS rates were similar between conditions [17]. Furthermore, and also aligned with the concerted global effort to advance sustainable human nutrition [25,26], recent studies have reported no clear association between the amplitude or rate of postprandial increase in blood leucine concentration and the magnitude of MPS response to the ingestion of alternative proteins in healthy young adults, including fungal-derived mycoprotein (*Fusarium venenatum*) [20,27,28], microalgae [19] or meal worm [29] derived insect protein. Taken together, these data highlight that ingested proteins that elicit a moderate postprandial leucine profile also exhibit the capacity to stimulate a robust increase in MPS, which may be explained (at least partially) via a leucine-independent mechanism(s).

**Table 1:** Summary of recent (2021 onwards) findings from studies that simultaneously measured blood leucine concentration profiles and postprandial rates of muscle protein synthesis in healthy young and older adults

Reference	Participants	Study design/intervention	Muscle fraction for postprandial MPS measurement	Evidence supporting “leucine trigger” hypothesis	Blood leucine concentration profile	Postprandial rates of MPS
Hermans *et al.* (2021)	24 healthy young (25 ± 4 y) males	Double-blind, parallel, RCT with 30 g intrinsically labeled mealworm-derived protein (WORM) vs. milk-derived protein (MILK) Unilateral bout of resistance exercise	Mixed MPS 0–5 h	No	Peak plasma leucine concentration: MILK > WORM by 27% (*P* < 0.001)	MILK (0.056 ± 0.012%· h^−1^) = WORM (0.045 ± 0.017%· h^−1^, *P* > 0.05) at rest MILK (0.073 ± 0.020%· h^−1^) = WORM (0.059 ± 0.015%· h^−1^, *P* > 0.05) postexercise
Hermans *et al.* (2022)	20 healthy young (25 ± 4 y) males	Parallel, RCT with 30 g cheese protein vs. milk protein concentrate Unilateral bout of resistance exercise	Mixed MPS 0–4 h	No	Peak plasma leucine concentration: Milk > cheese by 38% (*P* < 0.001)	Milk (0.056 ± 0.010%· h^−1^) = cheese (0.055 ± 0.018%· h^–1^, *P* > 0.05) at rest Milk (0.063 ± 0.010%· h^–1^) = cheese (0.067 ± 0.013%· h^–1^, *P* > 0.05) postexercise
Paulussen *et al.* (2023)	Healthy, physically active young (24 ± 4 y) males (*n* = 5) and females (*n* = 5)	Crossover, RCT with salmon (SAL) (20.5 g protein and 7.5 g fat) vs. constituent nutrients of salmon ingested in the form of crystalline amino acids, coconut oil, and fish oil (ISO)	MyoPS 0–5 h	No	AUC plasma leucine: SAL > ISO (*P* = 0.050)	SAL (0.056 ± 0.022% ·h^–1^) = ISO (0.046 ± 0.025%· h^–1^, *P* = 0.308)
Pinckaers *et al.* (2024a)	24 healthy young (24 ± 3 y) males	Double-blind, parallel, RCT with 30 g pea protein (PEA) vs. 30 g milk protein (MILK)	MyoPS 0–5 h		Peak plasma leucine concentration: MILK (353 ± 45 μmol /L) > PEA (282 ± 30 μmol/L ^−1^, *P* < 0.001) by ~25%	MILK (0.053 ± 0.013%·h^−1^) = PEA (0.053 ± 0.017%·h^−1^; *P* = 0.96)
Pinckaers *et al.* (2024b)	Healthy older (72 ± 4 y) males (*n* = 8) and female (*n* = 8) adults	Crossover, RCT with whole-food omnivorous meal containing beef as the primary source of protein (0.45 g protein/kg body mass; MEAT) vs. Isonitrogenous and isocaloric whole-food vegan meal (PLANT)	Mixed MPS 0–6 h		Peak plasma leucine concentration: MEAT (198 ± 23 μmol/L ^−1^) > PLANT (158 ± 21μmol/L ^−1^, *P* < 0.001) by ~25%	MEAT (0.052 ± 0.023%· h^–1^) >. PLANT (0.035 ± 0.021%· h^–1^, *P* = 0.037) by ~47%
Pinckaers *et al.* (2024c)	36 healthy young (26 ± 4 y) males	Double-blind, parallel, RCT with 30 g corn protein (CORN) vs. 30 g milk protein (MILK) vs. 30 g protein blend with 15 g corn plus 15 g milk protein (CORN+MILK)	MyoPS 0–5h		Peak plasma leucine concentration: CORN (390 ± 66 μmol/ L) = MILK (353 ± 45 μmol /L) = CORN+MILK (395 ± 62 μmol /L, *P* > 0.05) Time to peak leucine concentration: MILK (46 ± 43 min) < CORN (130 ± 35 min) and CORN+MILK (133 ± 45 min, all *P* < 0.001)	CORN (0.053 ± 0.013% ·h^–1^) = MILK (0.053 ± 0.013%· h^–1^) = CORN + MILK (0.052 ± 0.024, *P* > 0.05)
Pinckaers *et al.* (2023)	24 healthy young (24 ± 4 y) males	Double-blind, parallel, RCT with 30 g milk protein (MILK) vs. 30 g plant blend combining 15 g wheat, 7.5 g corn, and 7.5 g pea protein (PLANT-BLEND)	MyoPS 0–5h		Peak plasma leucine concentrations: MILK (353 ± 45 μmol /L ) > PLANT-BLEND (283 ± 22 μmol /L, *P* < 0.001) by ~25% Time to peak leucine concentration: MILK (46 ± 43 min) < PLANT-BLEND (113 ± 46 min, *P* = 0.001)	MILK (0.053 ± 0.013% ·h^–1^) = PLANT-BLEND (0.064 ± 0.016% ·h^–1^, *P* = 0.08)
Pinckaers *et al.* (2022)	24 healthy young (24 ± 4 y) males	Double-blind, parallel, RCT with 30 g potato-derived protein (POTATO) vs. 30 g milk protein (MILK) Unilateral bout of resistance exercise	Mixed MPS 0–5h		Peak plasma leucine concentrations: MILK (341 ± 65 μmol/ L) > POTATO (252 ± 23 μmol/L , *P* < 0.001) by 26% Postprandial plasma leucine availability: MILK (iAUC: 35 ± 8 μmol/L × 5h) > POTATO (27 ± 4 μmol/L×5h, *P* < 0.05) by 23% Time to peak leucine concentration: MILK (48 ± 27 min) < (153 ± 50 min, *P* < 0.001)	MILK (0.050 ± 0.012%· h^−1^), respectively; = POTATO (0.053 ± 0.017%·h^−1^, *P* = 0.540) at rest MILK (0.064 ± 0.015%·h^−1^) = POTATO (0.069 ± 0.019%·h^−1^, *P* = 0.520) postexercise
Pinckaers *et al.* (2021)	36 healthy (24 ± 3 y) males	Double-blind, parallel, RCT 30 g milk protein (MILK) vs. 30 g wheat protein (WHEAT) vs. 30 g blend combining 15 g wheat plus 15 g milk protein (WHEAT+MILK)	MyoPS 0–5 h		Plasma leucine increase was greater for MILK vs. WHEAT (time × treatment: *P* < 0.001) but did not differ between MILK and WHEAT+MILK (time × treatment: *P* = 0.09). Peak plasma leucine concentrations: MILK (353 ± 45 μmol /L) > WHEAT (280 ± 37 μmol/L, *P* < 0.001) and WHEAT+MILK (301± 44 μmol/L l, *P* = 0.01) Time to peak leucine concentration: MILK (46 ± 43 min) = WHEAT (58 ± 19 min, *P* = 0.42) = WHEAT+MILK: 64 ± 51 min, *P* = 0.31)	MILK (0.053 ± 0.013%· h^−1^) = WHEAT (0.056 ± 0.012%· h^−1^, *P* = 0.56) = WHEAT+MILK (0.059 ± 0.025%· h^−1^, *P* = 0.46)
van der Heijden *et al.* (2023)	Healthy young (22 ± 3 y males (*n* = 18) and females (*n* = 18)	Double-blind, parallel, RCT 25 g protein from fungal-derived from mycoprotein (MYCO) vs. Spirulina (SPIR) vs. Chlorella (CHLO) Unilateral bout of resistance exercise	MyoPS 0–4 h	No	Peak plasma leucine concentration: SPIR (316 ± 66 μmol/L) > MYCO (226 ± 30 μmol/L, *P* < 0.001) and CHLO (187 ± 30 μmol/L, *P* < 0.001)	MYCO (0.060 ± 0.015%·h^−1^) = SPIR (0.066 ± 0.022%·h^−1^) = CHLO (0.055 ± 0.019%·h^−1^, *P* > 0.05) at rest MYCO (0.092 ± 0.024%·h^−1^) = SPIR (0.086 ± 0.028%·h^−1^) = CHLO (0.090 ± 0.024%·h^−1^, *P* > 0.05) postexercise
Weijzen *et al.* (2022)	Healthy young (22 ± 3 y) males (*n* = 12) and females (*n* = 12)	Double-blind, parallel, RCT with 30 g intrinsically L-[1–^13^C]-phenylalanine–labelled milk protein (PRO) vs. 30 g of free amino acids (FAA) labelled with L-[1–^13^C]-phenylalanine	Mixed MPS 0–6 h	No	Peak plasma leucine concentration: FAA (501 ± 42 μmol /L ) > PRO (326 ± 59 μmol l/L, *P* < 0.001)	FAA (0.053 ± 0.014%·h^−1^) = PRO (0.051 ± 0.010%·h^−1^, *P* = 0.629)
West *et al.* (2023)	24 healthy young (21 ± 1 y) males	Double-blind, parallel, RCT with 70 g mycoprotein (MYC; 31.4 g protein, 2.5 g leucine) vs. 38.2 g of a protein concentrate obtained from mycoprotein (PCM; 28.0 g protein, 2.5 g leucine) Unilateral bout of resistance exercise	MyoPS 0–1.5 h	No	Peak plasma leucine concentration: PCM > MYC (*P* < 0·0001)	MYC = PCM at rest (*P* > 0.05) MYC = PCM postexercise (*P* > 0.05)
West *et al.* (2023a)	Healthy young (21 ± 1 y) males (*n* = 24) and females (*n* = 9)	Double-blind, parallel, RCT with 25 g of protein from mycoprotein (MYC) vs. pea protein (PEA) vs. blend (39% MYC, 61% PEA) of the two (BLEND) Whole-body bout of resistance exercise	MyoPS 0–4 h	No	Time to peak leucine concentration: PEA and BLEND > MYC (*P* < 0.0001)	MYC (0.076 ± 0.004%·h^−1^) = PEA 0.087 ± 0.01%·h^−1^) = BLEND (0.085 ± 0.01%·h^−1^; all *P* > 0.05)

AUC, area under curve; g, gram; h, hour; MPC, milk protein concentrate; MPS, muscle protein synthesis; Myo, myofibrillar; RCT, randomized controlled trial; y, years.

Values are presented as means ± SE.

Beyond individual constituents, a concerted effort has recently focussed on advancing understanding regarding the protein digestion, amino acid absorption and postprandial response of MPS to the ingestion of protein-rich whole foods [16,30] and mixed macronutrient meals [31], which is commonly termed a food-first approach. For instance, and highlighting the apparent disassociation between amino acid absorption kinetics and the postprandial stimulation of MPS, an elegant study by Paulussen *et al.* demonstrated comparable MPS rates with the postexercise ingestion of a 99 g salmon fillet *vs.* the same nutrients ingested as an isolated mixture of crystalline free amino acids and fish oil in resistance-trained young adults, despite a markedly earlier peak in EAA concentrations in the isolated constituent nutrients group. These data [16] are consistent with an earlier report whereby the consumption of skimmed milk stimulated a greater postprandial MPS response than the ingestion of minced beef, despite the observation that leucine availability was greater in the beef *vs*. milk condition [32]. Given that conditions were matched for protein content between studies, these data suggest that other components of the protein-rich food source beyond protein digestion and amino acid (leucine) absorption kinetics modulated the postprandial response of MPS. Hence, it could be argued that the role of postprandial leucine kinetics in regulating MPS may be less evident within the context of protein-rich whole food ingestion [14^▪^].

Alternative nutritional components of food (i.e., nutrient-nutrient interactions of protein with lipids, vitamins and minerals) or non-nutrient components of food related to form, preparation and processing has also been proposed to modulate the postprandial response of MPS, via distinct leucine-independent molecular mechanisms [33^▪^]. Aligned with the concept of a food matrix effect [2], the interaction of nutrients (e.g., protein, carbohydrates, fats, vitamins, minerals) contained in whole foods or nutrient-dense mixed meals are purported to be regulatory for the stimulation of MPS via multiple pathways. For instance, the carbohydrate content of a food source is primarily responsible for insulin secretion that facilitates the delivery of amino acids to skeletal muscle and subsequent activation of mTORC1 translocation and cell signalling [34]. Moreover, vitamins, minerals and other bioactive components of whole/mixed foodstuff also activate nutrient sensing mechanisms within the muscle cell that indirectly modulates the stimulation of MPS [33^▪^]. Nonnutrient components of food, specifically related to form, preparation and processing, are also implicated in modulating the postprandial MPS response via leucine-independent mechanisms [2]. For example, the ingestion of milk with higher glycation levels (induced via heat treatment) was shown to evoke an attenuated rise in plasma EAA concentrations (compared to lower glycation milk) which, in theory, would be rate-limiting for the postprandial stimulation of MPS [33^▪^]. Thus, while the anabolic action of leucine appears to be robust when provided via isolated protein sources, a leucine-independent effect on the regulation of MPS appears to be relevant when applied to more complex nutritional scenarios such as whole foods or mixed meals.

Interestingly, to our knowledge, the only recent study to demonstrate a positive association between blood leucine concentration profiles and the postprandial response of MPS to an ingested protein source was conducted in healthy older adults [21]. In this study, peak plasma leucine concentrations were 25% greater after ingesting a protein-matched (36 g) beef-based omnivorous meal *vs*. an isocaloric vegan meal that constituted plant-based whole foods, and was accompanied by a corresponding ~47% greater postprandial MPS response over the subsequent 6-h postprandial period [21]. These data are consistent with findings from two recent systematic reviews [3,4] that concluded the utility of the leucine trigger hypothesis in regulating the postprandial response of MPS appears to be age dependent in healthy adults. In this regard, a strong relationship (*r*^2^ = 0.64) was observed between the dose of ingested leucine and the postprandial response of MPS in healthy older adults, but this association was absent (*r*^2^ = 0.01) in healthy young adults [4]. The precise explanation for this apparent age-effect on the utility of the leucine trigger hypothesis is difficult to reconcile, but is possibly related to impairments in the absorption, transport and delivery of leucine to senescent skeletal muscle, coupled with a reduced sensitivity of intramuscular cell signalling proteins to leucine that is evident in healthy older adults [7]. Hence, the higher leucine threshold that is required to stimulate a postprandial response of MPS in older adults dictates that the rate of leucine appearance into the circulation features more prominently as both a limiting factor in activating the muscle protein synthetic machinery and in regulating the magnitude of postprandial MPS rates in ‘anabolic-resistant’ healthy older adults than ‘anabolic-sensitive’ healthy young adults. At the other end of the health spectrum, given that recent evidence indicates that amino acid absorption rates may be compromised in critical illness [5], intuitively the leucine trigger hypothesis may play a prominent role in devising amino acid/protein recommendations in this more compromised, albeit under-studied, clinical population. Accordingly, the remainder of this review summarizes the limited evidence base pertaining to protein digestion and amino acid absorption kinetics within an intensive care setting with a view to advancing clinical nutrition guidelines in critically ill patients.

## PROTEIN DIGESTION AND AMINO ACID ABSORPTION KINETICS IN CRITICALLY ILL PATIENTS

Intensive care poses an extreme challenge to critically ill patients whereby a rapid decline in skeletal muscle mass and function is associated with poor long-term functional recovery and increased mortality [6]. Indeed, critically ill patients are estimated to lose ~2% of their muscle mass per day following admission to intensive care which exacerbates risk of morbidity and mortality in this patient group [35,36]. The physiological mechanisms that underpin muscle atrophy in critical illness are not fully elucidated, but are clearly multifactorial with contributing features including elevated levels of undernutrition, inflammation, immobilization, hormonal alterations, and a muscle catabolic state underpinned by anabolic resistance. Gastrointestinal dysfunction is also common during critical illness [37] and some recent evidence indicates that postprandial amino acid absorption rates may be compromised among critically ill patients [5], although this observation is not universal. For instance, intravenous and enteral stable isotopic tracer studies reported a reduced systemic availability of phenylalanine in critically ill patients *vs*. healthy controls, although this observation may be ascribed to an impaired digestion rate or a higher splanchnic extraction of amino acids among critically ill patients [38]. Interestingly, another study reported an attenuated postprandial increase in EAA concentrations in critically ill patients which was not associated with small intestinal function [5]. Conversely, despite the enteral administration of a 20 g intrinsically-labelled milk protein eliciting comparable amino acid absorption kinetics between critically ill patients and healthy controls, Chapple *et al.* reported an ~60% reduction in the postprandial response of MPS in critically ill patients [39], thus reflecting a state of anabolic resistance to enteral protein provision. Hence, it seems intuitive that dietary-derived amino acid availability and the subsequent postprandial stimulation of MPS may be impaired in this more compromised, but under-studied, clinical population, although it remains unclear whether impaired digestion is a causal factor. Nonetheless, moving forward there is a need for experimental studies to establish optimal and pragmatic protein feeding strategies within a clinical setting of critical illness.

Accordingly, a recent proof-of-concept study investigated the acute (2-h) postprandial response of amino acid kinetics and whole-body net protein balance to manipulation of enteral amino acid intake in critical illness [40^▪▪^]. Specifically, van Gassel *et al.* compared the efficacy of enteral feeding via a nasogastric tube with 20 g of intrinsically-labelled intact milk protein *vs*. a custom made free amino acid mixture that contained an identical amino acid profile. The amino acid mixture condition elicited a more rapid and marked increase in systemic phenylalanine and leucine concentrations, and whole-body protein net balance tended to be more positive compared to the intact protein condition over a 2-h period in critically ill patients [40^▪▪^]. Whilst these preliminary data indicate that enteral administration of free amino acids may mitigate the catabolic response in critically ill patients, the high molarity of these products anecdotally leads to a high prevalence of diarrhoea which nullifies the clinical utility of free amino acids into clinical practice. Conversely, negligible side effects have been reported with semi-elemental products such as peptide-based diets. Moving forward, future studies that include direct tissue-specific, rather than whole-body, measurements of MPS are warranted to establish targeted and pragmatic protein or amino acid based interventions to mitigate skeletal muscle atrophy during critical illness (Table 2).

**Table 2:** Summary of recent (2022-) studies utilizing protein-based interventions in critically ill patients with functional and clinical endpoints

Reference	Participants	Study design	Intervention	Outcomes
Heuts *et al.* (2025)	*n* = 4164 patients aged ≥ 18 years	Systematic review and meta-analysis	Higher vs. lower protein groups (mean protein intake: 1.5 ± 0.6 vs. 0.9 ± 0.4 g/kg/day)	Risk ratio (RR) (1.01 (95% CI: 0.84–1.16) for mortality
Lee *et al.* (2024)	*n* = 3,303 aged ≥ 18 years	Systematic review and meta-analysis	Higher vs. lower protein groups (mean protein intake: 1.49 ± 0.48 g/kg/day vs. 0.92 ± 0.30 g/kg/day)	Risk ratio (0.99 (95% CI: 0.88–1.11) for mortality Self-reported quality-of-life physical function measurements at day-90 (Standardized mean difference → 0.40, 95% CI: –0.04–0.84) In patients with acute kidney injury: Mortality ↑ (RR 1.42; 95% CI: 1.11–1.82)
Qin *et al.* (2024)	*n* = 2,965 aged ≥ 18 years	Systematic review and meta-analysis	Higher (≥1.2 g/kg/day) vs. lower (<1.2 g/kg/day) doses of protein supplementation	RR (1.03; 95%CI: 0.92–1.15) for mortality Length of intensive care unit stay (0.19; 95% CI: -0.67–1.04) Length of hospital stay (0.73; 95%CI: -1.59–3.04) Duration of mechanical ventilation (-0.14; 95%CI: -0.83–0.54) RR (1.11; 95%CI: 0.87–1.41) for incidence of acute kidney injury
Araujo *et al.* (2024)	High group: *n* = 206; 60.3 ± 15.7 years Medium group: *n* = 325; 58.8 ± 14.3 years	Retrospective cohort study	Protein intake (g/kg/day): low (<0.8), medium (0.8–1.19), high (1.2–1.5), and very high (>1.5)	Protein dose 1.2–1.5 g/kg/day was associated with superior functional capacity at ICU discharge compared with other doses
van Gassel *et al.* (2024)	Protein group: *n* = 7; 55 (45, 60) years Amino acid group: *n* = 7; 71 (54, 73) years	Randomized controlled trial	20 g intrinsically L-[1–^13^C]-phenylalanine-labelled milk protein vs. 20 g intrinsically L-[1–^13^C]- phenylalanine amino acids for 6 h	Whole-body protein net balance became positive after nutrient administration (P-time < 0.001) and tended to be more positive after free amino acid in provision (P-time treatment = 0.07)
van Gassel *et al.* (2022)	*n* = 21; 47 (40, 60) years vs. 9 healthy controls 37 (19, 73) years	Non-randomized controlled trial	100 mL of a formula feed (Ensure) and 2 g of 3-*O*-Methyld-glucose (3-OMG) via postpyloric feeding tube for 60 to 240 min	Postprandial rise in essential amino acids was not apparent in critically ill patients compared with healthy controls (iAUC 60 min, −4858 [−6859 to 2886] vs 5406 [3099–16,853] μmol/L; *P* = 0.039). No significant differences were observed from 0–240 min
Chapple *et al.* (2022)	*n* = 15; 50 ± 17 years vs. 10 healthy controls 54 ± 23 years	Non-randomized controlled trial	Primed intravenous L-[ring-^2^H_5_]-phenylalanine, L-[3,5–2H_2_]-tyrosine, and L-[1–^13^C]-leucine infusion over 9.5 h and a duodenal bolus of intrinsically-labelled (L-[1–^13^C]-phenylalanine and L-[1–^13^C]-leucine) intact milk protein (20 g protein) over 60 min	Plasma amino acid availability did not differ between groups (ICU patients, 54.2 ± 9.1%, vs. healthy control subjects, 61.8 ± 13.1%; *P* = 0.12) Myofibrillar protein synthesis rates increased in both groups (0.028 ± 0.010%/h vs. 0.043 ± 0.018%/h with main time effect (*P* = 0.046), with lower rates in ICU patients vs. healthy control subjects (main group effect *P* = 0.001) Incorporation of protein-derived phenylalanine into myofibrillar protein was 60% lower in ICU patients (0.007 ± 0.007 mol percentage excess vs. 0.017 ± 0.009 mol percentage excess; *P* = 0.007)

## CONCLUSION

The relationship of protein digestion and amino acid absorption kinetics with the postprandial stimulation of MPS is complex. While the leucine trigger hypothesis provides invaluable mechanistic insights into the postprandial regulation of MPS, recent *in vivo* studies report a disassociation between blood leucine availability and postprandial MPS rates that is contingent upon physiological (i.e., healthy and clinical populations, rest and exercise) and nutritional (protein dose, isolated intact protein sources and protein-rich foods) factors. Preliminary evidence suggests that amino acid absorption kinetics are compromised in critically ill patients. Accordingly, future studies are warranted to determine best practice protein/amino acid-based guidelines to mitigate muscle atrophy during critical illness.

## Acknowledgements

*None.*

### Financial support and sponsorship

*None to declare.*

### Conflicts of interest

*O.C.W. has received honorarium from Danone and the Dairy Council UK for consultancy and conference speaking. C.S.D. and K.P. have none to report.*

## REFERENCES AND RECOMMENDED READING

Papers of particular interest, published within the annual period of review, have been highlighted as:

▪ of special interest
▪▪ of outstanding interest

## References

[1] Wolfe RR Church DD Ferrando AA Moughan PJ. Consideration of the role of protein quality in determining dietary protein recommendations. Front Nutr 2024; 13:1389664. 10.3389/fnut.2024.1389664 PMC11598328 39606577

[2] Barnes TM Deutz MT Zupancic Z. Protein quality and the food matrix: defining optimal versus maximal meal-based protein intakes for stimulating muscle protein synthesis. Appl Physiol Nutr Metab 2023; 48:340–344. 36735923 10.1139/apnm-2022-0373

[3] Zaromskyte G Prokopidis K Ioannidis T. Evaluating the leucine trigger hypothesis to explain the postprandial regulation of muscle protein synthesis in young and older adults: a systematic review. Front Nutr 2021; 8:685165. 34307436 10.3389/fnut.2021.685165 PMC8295465

[4] Wilkinson K Koscien CP Monteyne AJ. Association of postprandial postexercise muscle protein synthesis rates with dietary leucine: a systematic review. Physiol Rep 2023; 11:e15775. 37537134 10.14814/phy2.15775 PMC10400406

[5] van Gassel RJ van de Poll MC Schaap FG. Postprandial rise of essential amino acids is impaired during critical illness and unrelated to small-intestinal function. J Parenter Enteral Nutr 2022; 46:114–122. 10.1002/jpen.2103 PMC9293041 33666262

[6] Lees MJ Prado CM Wischmeyer PE Phillips SM. Skeletal muscle: a critical organ for survival and recovery in critical illness. Crit Care Clin 2025; 41:299–312. 40021281 10.1016/j.ccc.2024.08.011

[7] Gorissen SH Trommelen J Kouw IW. Protein type, protein dose, and age modulate dietary protein digestion and phenylalanine absorption kinetics and plasma phenylalanine availability in humans. J Nutr 2020; 150:2041–2050. 32069356 10.1093/jn/nxaa024 PMC7398787

[8] Trommelen J Tomé D van Loon LJ. Gut amino acid absorption in humans: concepts and relevance for postprandial metabolism. Clin Nutr Open Sci 2021; 36:43–55.

[9] Trommelen J Holwerda AM Pinckaers PJ van Loon LJ. Comprehensive assessment of postprandial protein handling by the application of intrinsically labelled protein in vivo in human subjects. Proc Nutr Soc 2021; 80:221–229. 33487181 10.1017/S0029665120008034

[10] Trommelen J van Lieshout GAA Nyakayiru J. The anabolic response to protein ingestion during recovery from exercise has no upper limit in magnitude and duration in vivo in humans. Cell Rep Med 2023; 4:101324. 38118410 10.1016/j.xcrm.2023.101324 PMC10772463

[11] Horstman AMH Huppertz T. Milk proteins: processing, gastric coagulation, amino acid availability and muscle protein synthesis. Crit Rev Food Sci Nutr 2023; 63:10267–10282. 35611879 10.1080/10408398.2022.2078782

[12] Tang JE Moore DR Kujbida GW. Ingestion of whey hydrolysate, casein, or soy protein isolate: effects on mixed muscle protein synthesis at rest and following resistance exercise in young men. J Appl Physiol 2009; 107:987–992. 19589961 10.1152/japplphysiol.00076.2009

[13] Holowaty MNH Lees MJ Abou Sawan S. Leucine ingestion promotes mTOR translocation to the periphery and enhances total and peripheral RPS6 phosphorylation in human skeletal muscle. Amino Acids 2023; 55:253–261. 36474017 10.1007/s00726-022-03221-w

[14▪] Monteyne AJ West S Stephens FB Wall BT. Reconsidering the preeminence of dietary leucine and plasma leucinemia for predicting the stimulation of postprandial muscle protein synthesis rates. Am J Clin Nutr 2024; 120:7–16. 38705358 10.1016/j.ajcnut.2024.04.032 PMC11251220

[15] Weijzen MEG van Gassel RJJ Kouw IWK. Ingestion of free amino acids compared with an equivalent amount of intact protein results in more rapid amino acid absorption and greater postprandial plasma amino acid availability without affecting muscle protein synthesis rates in young adults in a double-blind randomized trial. J Nutr 2022; 152:59–67. 34642762 10.1093/jn/nxab305 PMC8754581

[16] Paulussen KJ Barnes TM Askow AT. Underpinning the food matrix regulation of postexercise myofibrillar protein synthesis by comparing salmon ingestion with the sum of its isolated nutrients in healthy young adults. J Nutr 2023; 153:1359–1372. 36870539 10.1016/j.tjnut.2023.02.037

[17] Pinckaers PJM Smeets JSJ Kouw IWK. Postprandial muscle protein synthesis rates following the ingestion of pea-derived protein do not differ from ingesting an equivalent amount of milk-derived protein in healthy, young males. Eur J Nutr 2024; 63:893–904. 38228945 10.1007/s00394-023-03295-6 PMC10948472

[18] Pinckaers PJM Kouw IWK Hendriks FK. No differences in muscle protein synthesis rates following ingestion of wheat protein, milk protein, and their protein blend in healthy, young males. Br J Nutr 2021; 126:1832–1842. 33597056 10.1017/S0007114521000635

[19] van der Heijden I West S Monteyne AJ. Algae ingestion increases resting and exercised myofibrillar protein synthesis rates to a similar extent as mycoprotein in young adults. J Nutr 2023; 153:3406–3417. 37716611 10.1016/j.tjnut.2023.08.035 PMC10739781

[20] West S Monteyne AJ Whelehan G. Mycoprotein ingestion within or without its wholefood matrix results in equivalent stimulation of myofibrillar protein synthesis rates in resting and exercised muscle of young men. Br J Nutr 2023; 130:20–32. 36172885 10.1017/S0007114522003087 PMC10050220

[21] Pinckaers PJ Domić J Petrick HL. Higher muscle protein synthesis rates following ingestion of an omnivorous meal compared with an isocaloric and isonitrogenous vegan meal in healthy, older adults. J Nutr 2024; 154:2120–2132. 37972895 10.1016/j.tjnut.2023.11.004

[22] Pinckaers PJM Hendriks FK Hermans WJH. Potato protein ingestion increases muscle protein synthesis rates at rest and during recovery from exercise in humans. Med Sci Sports Exerc 2022; 54:1572–1581. 35438672 10.1249/MSS.0000000000002937 PMC9390237

[23] Pinckaers PJM Weijzen MEG Houben LHP. The muscle protein synthetic response following corn protein ingestion does not differ from milk protein in healthy, young adults. Amino Acids 2024; 56:8-z. 10.1007/s00726-023-03377-z PMC10844360 38315260

[24] Pinckaers PJM Kouw IWK Gorissen SHM. The muscle protein synthetic response to the ingestion of a plant-derived protein blend does not differ from an equivalent amount of milk protein in healthy young males. J Nutr 2023; 152:2734–2743. 36170964 10.1093/jn/nxac222 PMC9839989

[25] Morgan PT Carson BP Witard OC. Dietary protein considerations in a sustainable and ageing world: a narrative review with a focus on greenhouse gas emissions and skeletal muscle remodelling and maintenance. BMC Musculoskelet Disord 2024; 25:1030. 39702220 10.1186/s12891-024-07945-6 PMC11660970

[26] Smith K Watson AW Lonnie M. Meeting the global protein supply requirements of a growing and ageing population. Eur J Nutr 2024; 63:1425–1433. 38430450 10.1007/s00394-024-03358-2 PMC11329409

[27] Monteyne AJ Coelho MOC Porter C. Mycoprotein ingestion stimulates protein synthesis rates to a greater extent than milk protein in rested and exercised skeletal muscle of healthy young men: a randomized controlled trial. Am J Clin Nutr 2020; 112:318–333. 32438401 10.1093/ajcn/nqaa092

[28] West S Monteyne AJ Whelehan G. Ingestion of mycoprotein, pea protein, and their blend support comparable postexercise myofibrillar protein synthesis rates in resistance-trained individuals. Am J Physiol Endocrinol Metab 2023; 325:E267–E279. 37529834 10.1152/ajpendo.00166.2023 PMC10655824

[29] Hermans WJ Senden JM Churchward-Venne TA. Insects are a viable protein source for human consumption: from insect protein digestion to postprandial muscle protein synthesis in vivo in humans: a double-blind randomized trial. Am J Clin Nutr 2021; 114:934–944. 34020450 10.1093/ajcn/nqab115 PMC8408844

[30] Burd NA McKenna CF Salvador AF. Dietary protein quantity, quality, and exercise are key to healthy living: a muscle-centric perspective across the lifespan. Front Nutr 2019; 6:83. 31245378 10.3389/fnut.2019.00083 PMC6563776

[31] Domic J Pinckaers PJ Grootswagers P. A well balanced vegan diet does not compromise daily mixed muscle protein synthesis rates when compared with an omnivorous diet in active older adults: a randomized controlled cross-over trial. J Nutr 2025; 155:1141–1150. 39732437 10.1016/j.tjnut.2024.12.019

[32] Burd NA Gorissen SH van Vliet S. Differences in postprandial protein handling after beef compared with milk ingestion during postexercise recovery: a randomized controlled trial. Am J Clin Nutr 2015; 102:828–836. 26354539 10.3945/ajcn.114.103184

[33▪] Witard O Devrim-Lanpir A McKinley MC Givens DI. Navigating the protein transition: why dairy and its matrix matter amid rising plant protein trends. Nutr Res Rev 2025; 21:1–13. 10.1017/S0954422425000101 40254950

[34] Abdulla H Smith K Atherton PJ Idris I. Role of insulin in the regulation of human skeletal muscle protein synthesis and breakdown: a systematic review and meta-analysis. Diabetologia 2016; 59:44–55. 26404065 10.1007/s00125-015-3751-0

[35] Fazzini B Markl T Costas C. The rate and assessment of muscle wasting during critical illness: a systematic review and meta-analysis. Crit Care 2023; 27:2-0. 10.1186/s13054-022-04253-0 PMC9808763 36597123

[36] Hrdy O Vrbica K Kovar M. Incidence of muscle wasting in the critically ill: a prospective observational cohort study. Sci Rep 2023; 13:742–748. 36639540 10.1038/s41598-023-28071-8 PMC9839699

[37] Reintam Blaser A Preiser J Fruhwald S. Gastrointestinal dysfunction in the critically ill: a systematic scoping review and research agenda proposed by the Section of Metabolism, Endocrinology and Nutrition of the European Society of Intensive Care Medicine. Crit Care 2020; 24:224–224. 32414423 10.1186/s13054-020-02889-4 PMC7226709

[38] Liebau F Wernerman J van Loon LJC Rooyackers O. Effect of initiating enteral protein feeding on whole-body protein turnover in critically ill patients. Am J Clin Nutr 2015; 101:549–557. 25733640 10.3945/ajcn.114.091934

[39] Chapple LS Kouw IWK Summers MJ. Muscle protein synthesis after protein administration in critical illness. Am J Respir Crit Care Med 2022; 206:740–749. 35584344 10.1164/rccm.202112-2780OC

[40▪▪] van Gassel RJ Weijzen ME Kouw IW. Administration of free amino acids improves exogenous amino acid availability when compared with intact protein in critically ill patients: a randomized controlled study. J Nutr 2024; 154:554–564. 38103646 10.1016/j.tjnut.2023.12.015

